# Testing the effect of a dynamic descriptive social norm message on meat-free food selection in worksite cafeterias: a randomized controlled trial

**DOI:** 10.1186/s12916-025-04302-9

**Published:** 2025-08-12

**Authors:** Elif Naz Çoker, Rachel Pechey, Susan A. Jebb

**Affiliations:** 1 Nuffield Department of Primary Care Health Sciences, University of Oxford, Radcliffe Primary Care Building, Radcliffe Observatory Quarter, Woodstock Road, Oxford, OX2 6GG UK; 2 UCL Energy Institute, The Bartlett School of Environment, Energy, and Resources, University College London, 14 Upper Woburn Place, London, WC1H 0AE UK

**Keywords:** Social norms, Meat consumption, Randomized controlled trial, Behavioral intervention, Behavior change, Worksite cafeterias

## Abstract

**Background:**

Overconsumption of meat is a threat to planetary health. Meat consumption is socially and culturally patterned, and interventions using social norms could be a promising strategy to encourage meat reduction.

**Methods:**

We developed and tested the effectiveness of a dynamic descriptive social norm message displayed in worksite cafeterias (*N* = 25, intervention = 12, control = 13) to increase meat-free meal selection. The message was developed based on existing evidence and in collaboration with the catering company operating the cafeterias. The message communicated a specific change in target behavior, using a relevant and relatable referent group, grounding the desired behavior change in time and place, and included a clear call to action. The social norm messages were displayed in each intervention cafeteria for 8 weeks on free-standing banners, posters, and floor stickers. We compared the change in weekly percentage of meat-free meal sales (measured as number of meals sold) between intervention and control cafeterias through linear mixed-effects models. We conducted fidelity checks in intervention cafeterias and interviewed customers to assess perceptions of the intervention.

**Results:**

There was no evidence that the intervention led to an increase in sales of meat-free meals (− 2.22 percentage point change, 95% CIs [− 7.33, 2.90], *p* = 0.378). Pre-intervention baseline sales of meat-free meals varied by site, but there was no evidence the intervention was differentially effective for sites with higher vs. lower baselines. There was also no evidence that the intervention changed overall meal sales. The intervention was implemented with high fidelity, though out of 155 customers interviewed, 57% reported that they did not notice the messages, and only 2% correctly recalled the message.

**Conclusions:**

There was no evidence that empirically informed and co-created dynamic descriptive social norm messages increased selection of meat-free meals in worksite cafeterias. This could be due to low salience of the intervention in a busy, fast-paced environment, or the strength of existing eating habits in a workplace cafeteria. The findings suggest that norm messaging interventions, when delivered as an isolated intervention, may not be effective to change a complex and socially grounded dietary behavior such as meat consumption.

**Trial registration:**

OSF Registries, Registered September 23, 2022, https://osf.io/h7zkf

**Supplementary Information:**

The online version contains supplementary material available at 10.1186/s12916-025-04302-9.

## Background

Overconsumption of meat presents a threat to both the health of people and that of our planet [1]. Compared to plant-based protein sources, meat products emit significantly more greenhouse gas emissions [2], and livestock production uses considerably more land, requires large amounts of water, releases pollutants, and contributes to soil erosion [3]. The International Agency for Research on Cancer and World Health Organization have identified processed meat as a potential carcinogen and red meat as a likely carcinogen [4, 5], and excess meat consumption has also been linked to an increased risk of cardiovascular disease, colorectal cancer, and obesity [6, 7]. In the United Kingdom (UK), reported meat consumption has declined in recent years, but the average consumption of 86.3 g per day is still well above the targets for a sustainable diet which recommend 43 g per day of combined red meat and poultry consumption [8]. Curbing overproduction and consumption of meat is necessary for meeting climate change mitigation targets and improving public health [9].

Meat consumption is socially and culturally patterned, suggesting that targeting social norms could be a promising strategy to encourage meat reduction. Social norms refer to unwritten rules of society that specify what behaviors and attitudes are typical, expected, and/or approved in a particular social context [10]. While social norm interventions have been widely tested for changing other pro-environmental behaviors such as energy saving and recycling [11], and other eating behaviors like increasing fruit and vegetable intake [12], there is little evidence that communicating social norms is effective in reducing meat consumption [13].

Dynamic descriptive social norms refer to past changes and expected future trends in the approval and adoption of a given behavior [14, 15]. Using dynamic descriptive norms in interventions where the target is to discourage individuals from performing a commonly adopted behavior (e.g., choosing meat-based meals for lunch), communicates that an increasing proportion of people are moving away from the currently commonly performed behavior (e.g., are starting to choose plant-based meals for their lunch more often). There is some evidence that dynamic descriptive norm messages have effectively changed intentions in online surveys in the United States of America (USA), the Netherlands, Germany, and Italy [14–17]. However, a direct replication of one of the studies in Sparkman and Walton [15] in the UK provided no evidence of any effect on intentions to reduce meat consumption [18]. The effect of dynamic descriptive norm messaging has also been tested on meat consumption behavior, measured via changes in meal-purchase sales from various food settings, including university campus cafes in the USA, UK, and New Zealand [15, 19], an online lunch delivery website and fine-dining restaurant in the USA [20], department store restaurants in the UK [21], and an online supermarket in the Netherlands [22]. Out of these, only two of the four studies included in Sparkman, Weitz [20] found any effect on meat consumption. However, these previous studies have usually employed pre-post designs without matching controls [15, 17, 19], had one-off or very short periods of intervention exposure [15–17], tested interventions with majority student populations [15, 19], and had intervention materials that were not visually salient [21], warranting a need for more evidence.

The present study developed and tested the effectiveness of empirically informed dynamic social norm messages on the selection of meat-free meals in worksite cafeterias across the UK. Employing a randomized controlled trial (RCT) design, having an eight-week intervention period, targeting a diverse demographic of both office and manufacturing worksite cafeterias, and developing salient intervention materials, the study aimed to address the gaps identified in previous literature.

## Methods

### Study aims

The primary aim of the study was to assess the effect of the presence of dynamic descriptive social norm messages in the workplace cafeteria on the likelihood of cafeteria customers selecting a meat-free meal option. We hypothesized that the proportion of weekly plant-based meal sales would be higher in the intervention sites compared to the control sites.

We also hypothesized that the intervention would be more effective in cafeterias that served offices, had high plant-based meal availability, and high intervention fidelity. Our secondary aim was to assess whether the intervention had any unintended consequences, such as a decrease in revenue, that could decrease its acceptability by food retailers. We hypothesized that the intervention would have no impact on overall sales. Finally, we aimed to assess the salience and perceived credibility of the dynamic descriptive norm messages through opportunistic interviews with cafeteria customers. As these interviews were exploratory, we did not have a pre-specified hypothesis for their outcome.

### Study design

We conducted a two-arm, parallel, randomized controlled trial in worksite cafeterias with the assistance of the central management team of a large global catering company that provides contract catering services to various companies and institutions across the United Kingdom. The trial ran in 27 cafeterias for 8 weeks, starting on 10 October 2022 and ending on 03 December 2022.

The inclusion criteria required each cafeteria to (a) be based in the UK, (b) have electronic point-of-sale tills operated by the catering company, (c) be able to provide data at a detailed enough level to identify specific meals sold, and (d) offer hot main meal options (in line with the base menu for the catering company). The sample size was based on pragmatic factors, rather than a power calculation, including willingness of worksite cafeteria managers to join the trial, the size of the worksite they were serving, contracting company, and baseline meat-free meal sales. Cafeterias were initially invited to participate in the study via an email sent to them by the Head of Sustainability of the catering company and were put into contact with the research team once they expressed initial interest.

The cafeterias that took part in this study all had a daily two-hour lunch service during which hot main meals and sides, salad bar, sandwiches, wraps, beverages, and confectionery were available for purchase. Each cafeteria curated their selection of food based on their customer profiles by choosing from a standard list of recipes provided by the central management of the catering company. The hot meals differed daily, and customers could view a weekly menu in advance. The prices of the hot meals (both meat-based and meat-free) were similar to each other, with a maximum of 5–10 pence difference. All cafeterias were self-service, with customers walking through each station and choosing what they wanted to eat and paying for it at the tills before sitting down to consume the food. All cafeterias operated within a single company’s facility, only serving employees of that specific company.

After recruitment, eligible worksite cafeterias were randomized with an allocation ratio of 1:1 to one of the following conditions: control (no message) and intervention (social norm messages displayed prominently in the cafeteria). The lead author conducted the randomization via random number assignment using STATA version IC 16.1 [23], based on a random seed generated online (https://bit.ly/stata-random) to enable replication. The necessary code for the randomization sequence was written and run on a separate do-file, and the researchers did not view the numbers and conditions assigned to the cafeterias until the complete code was run. There was no stratification or limitation set for the process.

It was not possible to blind researchers or worksite cafeteria managers to the intervention allocation due to the nature of its implementation. However, cafeteria users were not made aware of the research during the trial period, and no explicit individual consent of cafeteria customers was obtained since no personally identifiable information was collected, and the data analyzed was at worksite-level. The catering company obtained verbal consent from the contracting companies where the worksite cafeterias were housed and from the cafeteria managers that expressed interest in joining the trial. Ethical approval was given by the University of Oxford Central University Research Ethics Committee (Reference: R72710/RE001).

### Intervention design and materials

**Fig. 1:**
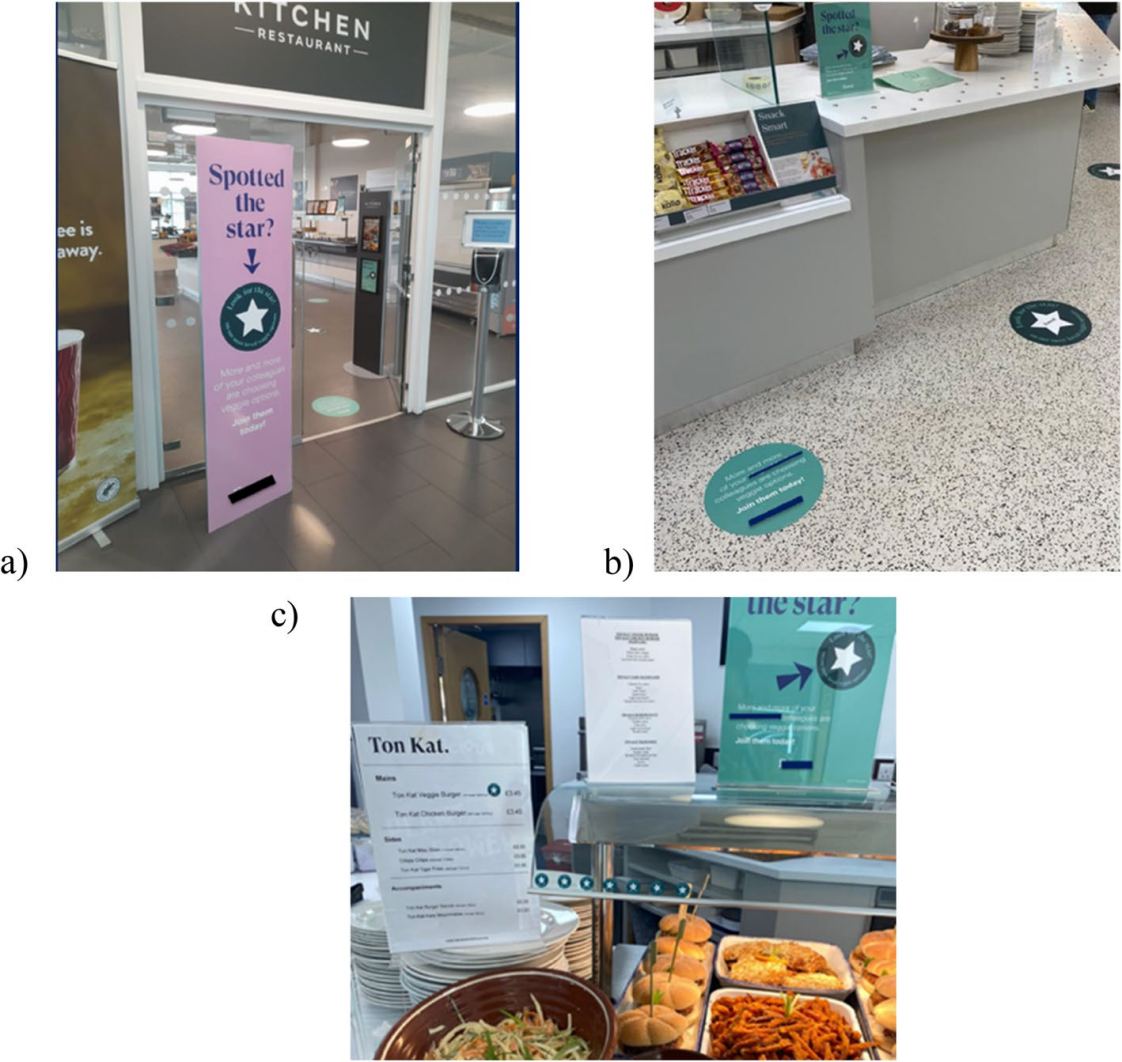
Intervention materials used in the study. **a** Large free-standing banner promoting the intervention message; **b** floor stickers placed in the dining area; **c** A4-sized posters with a star sticker indicating the vegetarian option on the menu. Company branding has been redacted for anonymity

The intervention consisted of three main components (see Fig. 1). The first component, “More and more of your [company name] colleagues are choosing veggie options,” conveyed the dynamic descriptive norm by implying an increased adoption of the target behavior (choice of vegetarian meal option). In line with evidence that higher context specificity increases the potential influence of social norms [24], the message referred to a specific and relevant peer groups, i.e., other co-workers of the contracting company in which the worksite cafeteria is located with the phrase “your [company name] colleagues.” The second component, “Join them today” aimed to reinforce the norm message with a call to action that invites the cafeteria customers to choose a plant-based meal “today,” grounding the performance of the normative behavior to a specific time [25, 26]. The final component, “Spotted the star? Look for the star on our most loved veggie options” added an element of gamification that urged customers to identify an easily perceivable feature (the star). It also highlighted the vegetarian dishes as the “most loved” option, which built on previous evidence that suggested drawing attention to plant-based dishes on menus by marking them as “dish of the day” or “chef’s recommendation” may increase their likelihood of getting selected [27].

The materials and visual aspects of the intervention were developed in collaboration with the marketing team and central management of the catering company. The materials consisted of a large free-standing three-sided banner placed at the entrance of the cafeteria, floor stickers that guided the customers from the entrance to the hot main meal buffet, A4-sized posters that were placed next to or above the buffet, and star stickers that were placed next to the vegetarian options on the menu. The banners, floor stickers, and posters all featured the three message components specified below and were all designed to make the messages as salient as possible by increasing exposure and catching attention (see Fig. 1).

### Procedure

All worksite cafeteria managers that provided verbal consent were invited to an online conference call where the research team outlined the trial design, purpose, and intervention materials. Following randomization, managers whose cafeterias were allocated to the intervention condition were invited to a second online call where researchers provided detailed information on how to implement the intervention and the procedure for fidelity checks.

Intervention cafeterias received their materials (i.e., banner, floor stickers, posters, menu star stickers) one week prior to the start of the trial and guidance on how to place the materials (banners at the entrance, floor stickers leading to hot buffet, and posters at the hot buffet) and marked the vegetarian options on their printout menus every day with the star stickers.

**Fig. 2:**
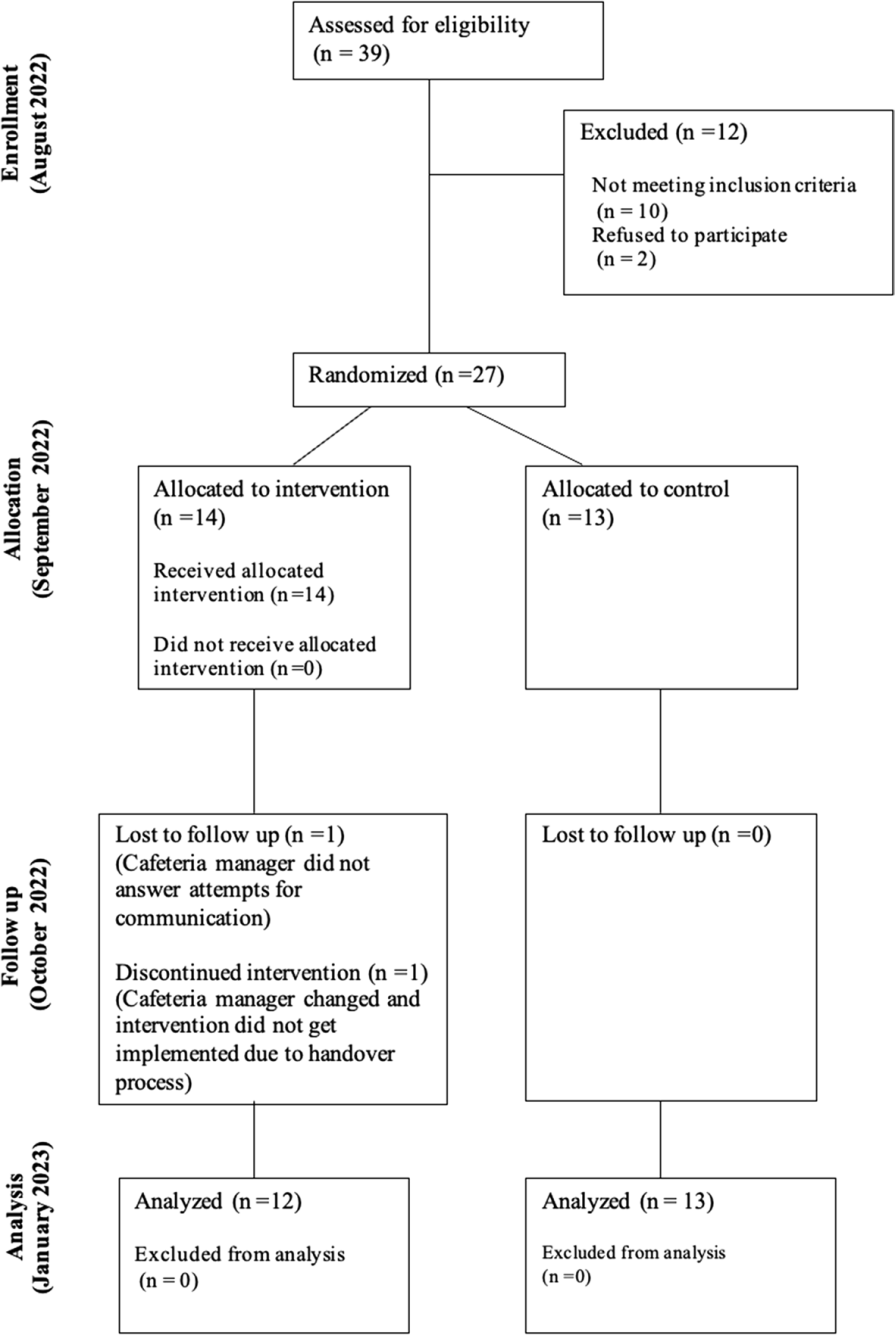
CONSORT flowchart illustrating the progression of cafeterias through each stage of the trial, including enrollment, allocation, follow-up, and analysis

All cafeterias, including those in the control condition, received four phone calls over eight weeks from researchers. All cafeterias were asked to report any site closures, till malfunctions, promotions and special events, unexpected changes to the preplanned menus, ingredient and supply shortages, or other disruptions and changes to the purchasing environment that could have affected footfall and sales. Intervention cafeterias were additionally asked whether the materials were still intact and on display, whether vegetarian options were being labelled daily, whether the catering staff had any feedback on the intervention, and whether any customers asked questions about the intervention materials. Intervention cafeteria staff were also required to send weekly photographs of the materials and menus to check that the intervention continued to be implemented correctly. One cafeteria manager was uncontactable, and one cafeteria had a new manager come into the role who did not receive information about the intervention during the handover. These two cafeterias were both part of the intervention condition and were treated as lost to follow up and excluded from analysis (Fig. 2).

During the final 2 weeks of the intervention, the research team visited 10 out of the 12 intervention cafeterias to observe the implementation in person and receive feedback from catering managers and staff. Team members arrived at each cafeteria shortly before lunch service and observed the customers’ arrival to the cafeteria, their food purchasing patterns, and their interactions during consumption. Opportunistic semi-structured interviews with willing cafeteria customers were conducted, who were asked whether they noticed the intervention materials, whether they could recall the content of the messages, whether they found this content to be credible and believable, whether they had a positive or negative reaction to the messages, and whether they made changes in their consumption pattern as a result. Team members recorded answers in writing using questionnaire templates (see Additional File 1: S1).

### Measures

#### Meal sales

The catering company provided the research team with sales data that were recorded via electronic point-of-sale tills throughout the trial. Hot main meals, wraps, salads, soups, sandwiches, savory snacks, starters, and jacket potatoes were coded as either meat-based or meat-free depending on their ingredients using a series of keywords (e.g., “chicken,” “fish,” “lamb” or “Quorn,” “plant-based,” vegan”). Food items that do not constitute a meal such as sides, confectionery, desserts, beverages, and condiments were excluded from the data. All products were individually checked by a member of the research team for potential errors in coding (e.g., coding a meat-including item as meat-free). Clarifications were asked from catering managers if the contents of a dish were not clear from its name. Following coding, the total number of meals sold every day and every week at each site were calculated, for both the 8-week period that preceded the trial which constituted the baseline, and the eight-week period of the trial itself. The total number of meals was used as a proxy to understand whether there were changes in footfall and overall meal selection in cafeterias during the intervention period. The proportion of meat-free dish sales was calculated for each day and each week for all participating worksite cafeterias for baseline and trial periods, which was the main outcome of interest.

#### Baseline meal sales

The catering company provided the research team with sales data for the 8-week period that preceded the intervention. Baseline meal sales were entered into all regression models as a control variable.

#### Worksite type

This was used as a proxy to estimate the socioeconomic status of the cafeteria customers, with offices assessed as having a larger concentration of higher SES customers and factories/manufacturing sites as having a larger concentration of lower SES customers. Worksites were classified as an office site if their main activities included financial, accounting, and customer services and telecommunications, and as a manufacturing site if they focused on production, processing, distribution, and storage of various goods.

#### Intervention fidelity

Each site was coded for its intervention fidelity based on its adherence to the intervention period based on data gathered from phone calls, photographs, and cafeteria visits. Cafeterias that displayed the materials correctly throughout, answered all phone calls, and provided weekly photos were coded to have high intervention fidelity, whereas those that missed one or more calls or did not send photos each week were coded to have low fidelity, creating a dummy variable.

#### Cafeteria closures

Any closures or unexpectedly low footfall days due to bank holidays, worker strikes, or worksite-specific events were recorded, and observations from those days were excluded from the data.

#### Till malfunctions

Any issues with recording sales using the electronic point of sale devices (e.g., due to internet connection failures or malfunctioning devices) were recorded for each cafeteria, and observations from those days were excluded from the data.

### Statistical analysis

The statistical analysis plan specifying hypotheses and primary and secondary outcomes was pre-registered on OSF before any data cleaning or analysis was conducted (https://osf.io/6wfgu). The analysis was done using STATA version IC 16.1 [23]. The data received from the catering company were cleaned, and discrepancies in records from each cafeteria (e.g., different ways of entering sale dates) were resolved prior to any analysis. Non-meal food items (e.g., confectionery—as explained in the Measures section above) were excluded from the data. Observations from weekends and national holidays were excluded from the data as they only occurred in a small number of sites and had a very low number of observations. Days where cafeterias experienced a till malfunction, closure, or strikes were excluded from the data, and a variable that indicated that the cafeteria had a shorter week was created. Overall, 7 intervention and 5 control cafeterias experienced at least one day of till malfunction, closure, or strikes, resulting in a total of 47 baseline period days/cafeteria (out of 1425 days/cafeteria) and 58 days/cafeteria (out of 1375 days/cafeteria) being excluded from the final analysis.

The primary research question was whether the presence of dynamic descriptive social norm messages in the workplace cafeteria changed the likelihood of cafeteria customers selecting a meat-free meal option, when controlling for baseline percentage of vegetarian main meal sales and length of operational week. Following from this, we also explored whether the effect of the presence of dynamic descriptive social norm messages in the workplace cafeteria varied when interacted with baseline sales, worksite type, and intervention fidelity. To answer these questions, multilevel mixed-effects linear regressions with restricted maximum likelihood and with Kenward-Roger method for degrees of freedom were run with autoregressive (AR) structure of order for within-group errors where the weeks were used as an integer-valued time variable to order the observations within groups and to determine the lags between successive observations. The p-value thresholds for significance were set at 0.05 for the primary and at 0.01 with a Bonferroni correction for multiple testing for the secondary research question. The weekly percentage of meat-free sales during the intervention period was first regressed on trial arm (control (*N* = 13) versus intervention (*N* = 12)), entering baseline percentage of meat-free meal sales and length of operational week as covariates. Then, another regression model was created adding type of worksite (office versus manufacturing) and baseline percentage of meat-free meal sales as interaction terms, with random effects varying according to each cafeteria. The regressions were repeated with a per-protocol analysis excluding 2 cafeterias from the intervention arm that had low intervention fidelity for a sensitivity analysis.

The secondary research question examined whether the overall sales of meals by the cafeterias changed with the presence of dynamic descriptive social norm messages. For this question, the above regression model was used again, but the dependent variable was replaced with weekly total meal sales during the intervention period, and the baseline variable was calculated based on total meal sales. The regression was repeated with a per-protocol analysis excluding 2 cafeterias from the intervention arm that had low intervention fidelity for a sensitivity analysis.

We also explored how cafeteria customers perceived the dynamic descriptive social norm messages in the workplace cafeteria. For this question, opportunistic interviews with cafeteria customers were descriptively analyzed for the frequency of recalls of the intervention material and message, the number of customers who thought the message was believable and credible, whether the message was able to motivate individuals to change their behavior, and the priorities of individuals when choosing a meal at their worksite cafeteria. These interviews took place during the last 2 weeks of the intervention trial period during regular lunch service hours when customers were most likely to visit the cafeterias.

The current analysis deviated from the original statistical analysis plan in several aspects. We initially intended to limit our analysis to hot main meal sales in each cafeteria, but due to the low sales of these items and the comparatively high sales of wraps, sandwiches, salads, and pasties in each cafeteria, the scope of the sales data which we analyzed was extended. The original plan also aimed to assess the availability of meat-free hot main meals in each cafeteria and enter this as an interaction variable to predict the effect of the intervention on meat-free sales. However, both due to the inclusion of other food items in the analyzed data and the lack of consistent information about the content of menus offered at each cafeteria, we decided that it was not possible to meaningfully measure the availability of meat-free items. Finally, the original plan intended to assess the change in the environmental impact of meals sold as a result of the intervention. However, since there was no evidence of a main effect of the intervention on meal sales, we did not go ahead with this analysis.

## Results

### Descriptive analyses

Two of 14 intervention cafeterias were excluded from the analysis as they were lost to follow-up. Nine out of the remaining 12 intervention cafeterias served offices, whereas three served manufacturing sites. Five out of 13 control cafeterias served offices, and eight served manufacturing sites. There was considerable variation across cafeterias in baseline weekly total number of meal sales (range, 97.8–3282.3) and percentage of weekly meat-free meal sales (range, 9.5–42.6%) (see Tables 1 and 2).

**Table 1:** Mean weekly total meal sales (measured as number of meals sold) and mean weekly percentage of meat-free meal sales for each trial arm during the baseline and trial periods

**Trial arm**	**Weekly total meals sold (SD)**		**Weekly percentage of meat-free** **meal sales % (SD)**	
	**Baseline**	**Trial**	**Baseline**	**Trial**
**Intervention**	343.12 (235.26)	386.29 (218.55)	27.73% (6.83)	27.31% (5.58)
**Control**	716.81(885.15)	781.87 (946.20)	24.86% (10.78)	24.87% (10.25)

**Table 2:** Baseline mean weekly total meal sales, baseline mean weekly percentage of meat-free meal sales, trial period mean weekly total meal sales, trial period mean weekly percentage of meat-free meal sales, trial arm, worksite type, and intervention fidelity of each cafeteria that participated in the study

Cafeterias	Trial arm	Worksite type	Baseline period mean weekly total meal sales (SD)	Baseline period mean weekly % of meat-free meal sales (SD)	Intervention fidelity^1^	Trial period mean weekly total meal sales (SD)	Trial period mean weekly % of meat-free meal sales (SD)
Cafeteria 1	Intervention	Office	133 (24.10)	19.48 (2.48)	High	180.75 (15.29)	21.58 (5.43)
Cafeteria 2	Intervention	Office	250.5 (45.79)	19.26 (3.69)	High	305.13 (36.26)	27.78 (2.67)
Cafeteria 3	Intervention	Office	262.13 (33.09)	30.22 (4.94)	High	336.38 (67.15)	28.54 (3.61)
Cafeteria 4	Intervention	Manufacturing	689.63 (106.12)	36.47 (4.61)	High	816.38 (41.80)	34.78 (3.00)
Cafeteria 5	Intervention	Manufacturing	243.13 (22.44)	29.59 (3.97)	High	279.38 (38.00)	25.80 (2.12)
Cafeteria 6	Intervention	Manufacturing	396.75 (75.53)	25.10 (6.21)	High	406.75 (43.17)	24.74 (2.00)
Cafeteria 7	Intervention	Manufacturing	165.63 (15.07)	27.01 (6.74)	High	215.5 (60.08)	21.35 (5.79)
Cafeteria 8	Intervention	Office	404.25 (71.80)	33.16 (3.66)	Low	437.13 (70.31)	31.96 (2.86)
Cafeteria 9	Intervention	Office	214.63 (47.87)	29.59 (2.66)	High	263.25 (50.02)	29.58 (3.58)
Cafeteria 10	Intervention	Office	275.38 (60.69)	29.38 (4.48)	High	333.13 (40.85)	32.77 (2.73)
Cafeteria 11	Intervention	Office	186.50 (27.80)	20.69 (5.34)	High	212.25 (26.50)	20.77 (4.18)
Cafeteria 12	Intervention	Office	895.88 (237.62)	32.78 (2.50)	Low	849.50 (58.52)	28.12 (2.82)
Cafeteria 13	Control	Office	3283.3 (307.32)	42.62 (3.83)	N/A	3524.38 (191.48)	39.30 (1.78)
Cafeteria 14	Control	Manufacturing	193.13 (44.24)	9.45 (5.52)	N/A	199.25 (30.52)	10.25 (2.82)
Cafeteria 15	Control	Office	342.63 (50.26)	21.21 (3.84)	N/A	385.00 (35.63)	29.75 (2.75)
Cafeteria 16	Control	Office	1635.9 (224.55)	23.96 (2.06)	N/A	1811.00 (87.95)	23.59 (1.40)
Cafeteria 17	Control	Manufacturing	226.50 (44.08)	18.62 (3.88)	N/A	245.00 (26.78)	16.17 (2.83)
Cafeteria 18	Control	Manufacturing	160.38 (61.91)	41.55 (7.63)	N/A	222.38 (36.02)	41.95 (4.62)
Cafeteria 19	Control	Manufacturing	506.13 (106.82)	24.61 (4.67)	N/A	454.13 (58.11)	23.66 (2.57)
Cafeteria 20	Control	Office	120.25 (31.14)	33.67 (8.92)	N/A	144.88 (24.91)	31.92 (7.83)
Cafeteria 21	Control	Office	848.50 (138.18)	19.34 (3.32)	N/A	939.50 (120.69)	22.93 (5.53)
Cafeteria 22	Control	Manufacturing	1283.8 (257.43)	21.51 (1.89)	N/A	1413.25 (154.21)	19.44 (1.41)
Cafeteria 23	Control	Manufacturing	97.75 (14.36)	26.74 (6.97)	N/A	108.88 (12.57)	22.60 (11.63)
Cafeteria 24	Control	Manufacturing	229.25 (44.98)	14.22 (10.79)	N/A	264.00 (43.69)	14.89 (9.69)
Cafeteria 25	Control	Manufacturing	391.13 (54.95)	25.65 (3.32)	N/A	452.63 (29.95)	26.90 (3.76)

^1^Each site was coded for their intervention fidelity based on their adherence to the intervention period based on data gathered from phone calls, photographs and cafeteria visits. Cafeterias that displayed the materials correctly throughout, answered all phone calls, and provided weekly photos were coded to have high intervention fidelity, whereas those that missed one or more calls or did not send photos each week were coded to have low fidelity, creating a dummy variable

### Effect of the intervention

There was no significant effect of the intervention on the percentage of weekly meat-free meal sales (− 2.22 percentage points, 95% CIs [− 7.33, 2.90], *p* = 0.378), controlling for length of operational week and baseline meat-free sales (Table 3).

**Table 3:** Mixed-effects regression model results for weekly percentage of meat-free meal sales during the intervention period

Variables	Model 1					Model 2				
	Model estimate^*^	**Standard error**	**95% CIs**	***t***	***p*-value**	**Model estimate**	**Standard error**	**95% CIs**	***t***	***p*-value**
	**Fixed effects**					**Fixed effects**				
(Intercept)	22.64	1.65	[19.22 26.05]	13.73	0.000	20.31	1.86	[16.42, 24.20]	10.91	0.000
Trial Arm^1^ (ref = Control)	− 2.22	2.47	[− 7.33, 2.89]	− 0.90	0.378	2.54	3.91	[− 5.66, 10.73]	0.65	0.525
Length of Week^2^ (ref = 5 Days)	− 1.24	1.09	[− 3.39, .92]	− 1.13	0.258	− 1.10	1.09	[− 3.26, 1.06]	− 1.01	0.316
Baseline meat-free sales^3^ (ref = Low)	10.62	2.48	[5.47, 15.76]	4.28	0.000	15.52	3.52	[8.16, 22.88]	4.41	0.000
**Intervention x High Baseline**^**4**^						− 9.94	4.71	[− 19.80, − 0.09]	− 2.11	0.048
Cafeteria Worksite Type^5^ (ref = Manufacturing)						3.04	3.05	[− 3.35, 9.43]	1.00	0.331
**Intervention x Office**^**6**^						− 1.68	4.36	[− 10.81, 7.46]	− 0.38	0.705
	**Random effect**					**Random effect**				
(Intercept)	27.44	9.27	[14.16, 53.20]			22.49	8.35	[10.86, 46.58]		

^*^Percentage point change in meat-free meal sales

^1^Trial arm refers to whether a cafeteria was randomized to the control or intervention condition during the RCT

^2^Length of week refers to the number of days the cafeteria was open and operational. Some cafeterias experienced closures due to strikes and till malfunctions which led them to be open 4 days of the week instead of five, and this was entered to the model as a dummy variable

^3^Baseline meat free meal sales refer to whether a cafeteria had low (i.e., lower than the mean percentage of meat-free meal sales of all cafeterias) or high (i.e., higher than the mean percentage of meat-free meal sales of all cafeterias) percentage of meat-free meal sales during the eight-week period prior to the trial

^4^This interaction was entered into the model to predict the effect of a cafeteria that had a high baseline of meat-free meal sales being randomized into the intervention arm of the trial on its percentage of meat-free meal sales during the trial period

^5^Cafeteria worksite type refers to the nature of work being performed in the specific worksite that each cafeteria service. Worksites are either categorized as manufacturing (e.g., production and processing of various goods, distribution and storage facilities) or office (e.g., customer service call centers, financial and accounting services) sites

^6^This interaction was entered into the model to predict the effect of a cafeteria that was serving a worksite that was categorized as an office site being randomized into the intervention arm of the trial on its percentage of meat-free meal sales during the trial period

There was no evidence that the effectiveness of the intervention varied in relation to the type of worksite (office versus manufacturing, 3.04 percentage point difference; 95% CIs, [− 3.35, 9.43]; *p* = 0.331) or baseline sales (high versus low baseline vegetarian sales, − 9.94 percentage point difference; 95% CIs, [− 19.79, − 0.09]; *p* = 0.048) at the pre-specified threshold for statistical significance, but this analysis is potentially underpowered due to the sample size (Table 3).

There was no evidence of an effect of the intervention on the weekly total number of meal sales across sites, controlling for length of week and baseline total sales (− 223 meals; 95% CIs, [− 635, 188]; *p* = 0.273) (see Additional File 1: Table S2).

Intervention fidelity was high, with only two out of twelve intervention cafeterias rated as low; so in place of a regression analysis, we conducted a sensitivity analysis excluding these sites. This did not alter the primary findings (see Additional File 1: Tables S3-5).

### Customer perceptions

Across 10 sites, 155 cafeteria customers participated in opportunistic interviews. Most (*n* = 88, 57%) customers did not recall seeing the intervention materials. Of those who could recall seeing the materials (*N* = 67, 43%), only three people were able to recall the dynamic norm message correctly. Around a quarter (*N* = 19, 28.4%) of those who saw the materials recalled it contained the message “Spotted the star?” and a similar proportion (*N* = 18, 26.9%) recalled it contained information about vegetarian options. Only five people recalled that the message both referred to a star and vegetarian options. The component that was recalled the most was the star stickers that were placed next to the meat-free options on the physical menus on display on the hot meal buffet. Among 67 customers who recalled seeing the materials, only three of them said that it encouraged them to change their lunch choices. Of those who recalled seeing the messages, 50 of them answered the question of whether they found the message to be believable. Sixty-two percent (*N* = 31) said “yes” and mentioned they have observed their colleagues choosing more meat-free options in the recent past. Foty-four customers responded to the question regarding their priorities when choosing their lunch options at the cafeteria. Forty-three percent (*N* = 19) mentioned taste and visual appeal as their priority. Value for money was mentioned by four customers, while health was mentioned by two and sustainability by none as an important factor in food choices.

## Discussion

A randomized controlled trial testing the effects of a dynamic descriptive norm message intervention on the selection of meat-free meal choices in worksite cafeterias in the UK found no evidence of an effect of the intervention.

For this study, we collaborated with a global catering company that operates a large number of cafeterias in the UK, which allowed for the testing of a social norm messaging intervention to reduce meat sales in a real-world setting, targeting a range of worksites (both office-based and manufacturing) located across England. We tested the intervention using a robust RCT design and were able to objectively measure meat consumption behavior through food purchasing data. Additionally, the creative aspects of message delivery (i.e., the visual design, size, positioning, and material and the wording of the social norm message) was developed in consultation with the catering company’s nutrition and marketing teams, drawing from their consumer insights, commercial expertise, and data-based knowledge of food purchasing trends across their worksites. Through this collaboration, the study contributed to meeting a need demonstrated by previous research for cooperation between psychology and nutrition science research and input from the food industry and retailers [28].

The study also had a few limitations. First, we were unable to assess any individual level differences in cafeteria customers. For example, we cannot know whether the intervention had an effect on some individuals such that it increased their selection of meat-free meals, and whether it backfired on others such that they showed a reactance to the intervention and selected more meat-based meals, resulting in an overall lack of effect. Secondly, we followed a non-stratified randomization strategy, which resulted in office cafeterias being overrepresented in the intervention condition (66%) compared to the control condition (38%). Linked to this, there were also discrepancies between cafeterias in terms of the percentage of the workforce who came into work in person and used the cafeteria regularly, and those who worked in a hybrid model and only used the cafeteria once or twice per week. The intervention also took place during a period where a number of worksites went through closures due to industry strikes, which meant that the cafeterias of these worksites were operational for fewer days. This could have potentially created differences in the frequency and duration of exposure to intervention materials. We accounted for this in our analyses but found no change in the effect of the intervention. These factors could have also weakened the relevance and salience of the referent group of “other colleagues” as the employees experienced disruptions in the number of days they spent time at the worksite and with their colleagues, which could have potentially decreased group cohesion and perceptions of shared identity, and impacted the effectiveness of the norm message [29]. Thirdly, while the cafeterias were operated by the same catering company and had comparable menu offerings, pricing, and marketing policies, they also served different companies and organizations. This meant that cafeterias differed not only in size and layout but also in terms of the clientele they serve and the company culture they exist in, evidenced by very different baseline uptakes of meat-free options and volume of overall sales.

The trial also accounted for a number of factors that could have impacted the effectiveness of the intervention. While the intervention message provided normative information about others’ behaviors, we anticipated that this could be either supported or hindered by which types of dishes cafeteria customers observed their colleagues choose in real life [30]. We therefore collected data on sales of meat-free meals for the eight-week period prior to the intervention and found that baseline sales did not impact the effect of the intervention. Previous field studies have had issues with intervention fidelity [21]. We measured intervention fidelity of each site at various time points and found that only two out of 12 sites had imperfect implementation. Excluding these two sites from the analysis did not change the outcome of the trial. Previous research has found that people who have lower socioeconomic status are more likely to overconsume meat, while those with higher socioeconomic status are more likely to have tried and/or consumed meat alternatives [31]. We used type of worksite as a proxy for socio-economic status to explore whether implementing the intervention in cafeterias that mainly served office workers or in those that mainly served manufacturing workers made a difference in its effectiveness and found no differences between the two worksite types.

This study adds to the small, but growing body of literature which has shown that while dynamic descriptive norms may be effective in changing self-reported intentions to reduce meat consumption, they might not be sufficient to change real-world behavior. For example, while effective in a campus café and a restaurant lunch setting in the USA, dynamic descriptive norm messages did not change meat-free meal sales in an online lunch delivery website or at a restaurant dinner setting [15, 20]. Dynamic descriptive norm messaging has also been found to be ineffective in changing meat consumption behavior across department store restaurants in the UK [21]; in university cafes in the UK and New Zealand [19]; and in an online supermarket in the Netherlands [22]. While previous and more established body of evidence indicate that perceptions of social norms are an important determinant of eating behavior [12, 32] these findings suggest that they might not be an effective mechanism for behavior change interventions to reduce meat consumption.

Despite the efforts made to formulate the dynamic descriptive norm message in line with existing social psychology theory and to make the intervention materials highly visible and salient in this trial, most customers who were interviewed did not recall seeing the materials. Interventions that attempt to relay normative information through text-based communication depend heavily on individuals to spend time and cognitive effort on reading, understanding, internalizing, and acting on the information. In fast-paced decision-making food purchasing settings, few individuals have the opportunity to pause, notice, and reflect on the intervention message. The short-term messaging interventions could also have been insufficient to challenge existing norms that repeat customers of the cafeterias have formed through multiple observations of colleagues’ food choices over long periods of time.

The relative ineffectiveness of social norm interventions to change meat-eating behavior compared to some other dietary habits may be because meat consumption is more complex and socially and culturally charged than other food consumption behaviors. Meat has historically been associated with ideas of masculinity [33], affluence [34], natural hierarchy [35], religiosity [36], and kinship [37]. The symbolic values assigned to meat consumption make it a particularly challenging behavior to change through communicating normative information, unlike other food behaviors such as increasing fruit and vegetable intake or choosing healthier snacks that have been successfully changed with norm-based interventions [38–40].

While dynamic descriptive social norm message-based interventions may not be effective on their own for reducing meat consumption, we cannot exclude the possibility that they could be effective when combined with other interventions that implicitly indicate what other consumers prefer, such as increasing the availability of meat-free meal options [41, 42], making meat-free options the default choice [43], or placing these options in more prominent positions [44, 45]. Future research can explore whether adding a norm message to these other proven interventions can amplify their effects and motivate greater reductions in meat consumption.

## Conclusions

A dynamic descriptive social norm message intervention did not increase meat-free meal selection in worksite cafeterias. The fast-paced and overstimulating food purchasing setting of the worksite cafeterias, the potential for cognitive exhaustion alongside hedonic preferences of the customers, and the sociocultural importance and symbolic value of meat consumption may have contributed to this ineffectiveness.

## Supplementary Information

Additional file 1: S1-S5 S1. Script for opportunistic interviews with cafeteria customers. S2. Mixed-effects regression model results for weekly total meal sales during the intervention period. S3. Sensitivity analysis (conducted excluding two cafeterias with low intervention fidelity) mixed-effects regression model results for weekly percentage of meat-free meal sales during the intervention period. S4. Sensitivity analysis (conducted excluding two cafeterias with low intervention fidelity) mixed-effects regression model results for weekly percentage of meat-free meal sales during the intervention period with interactions S5. Sensitivity analysis (conducted excluding two cafeterias with low intervention fidelity) mixed-effects regression model results for weekly total meal sales during the intervention period.

Additional file 2:  Completed CONSORT checklist

## Data Availability

The datasets generated during and/or analysed during the current study are not publicly available but can be made available from the corresponding author on reasonable request and through expressed written permission from the collaborating catering company.

## Abbreviations

UK: United Kingdom
US: United States
RCT: Randomized controlled trial
