# Supplementary material for: Testing the effect of a dynamic descriptive social norm message on meat-free food selection in worksite cafeterias: a randomized controlled trial
Source: BMC Med. 2025 Aug 12;23:474. doi: 10.1186/s12916-025-04302-9 (PMC12345364; doi:10.1186/s12916-025-04302-9)
Supplement: Supplementary file 1 — Additional file 1: S1-S5 S1. Script for opportunistic interviews with cafeteria customers. S2. Mixed-effects regression model results for weekly total meal sales during the intervention period. S3. Sensitivity analysis (conducted excluding two cafeterias with low intervention fidelity) mixed-effects regression model results for weekly percentage of meat-free meal sales during the intervention period. S4. Sensitivity analysis (conducted excluding two cafeterias with low intervention fidelity) mixed-effects regression model results for weekly percentage of meat-free meal sales during the intervention period with interactions S5. Sensitivity analysis (conducted excluding two cafeterias with low intervention fidelity) mixed-effects regression model results for weekly total meal sales during the intervention period. [file 12916_2025_4302_MOESM1_ESM.docx]

**Supplementary Materials**

**S1.** Script for opportunistic interviews with cafeteria customers.

1. Have you noticed any new informative messages in the cafeteria in the past two months?
   1. Yes
   2. No
2. If yes, what did the message say?
3. Did this message have an effect on your lunchtime meal choices?
4. Yes, please say what effect this message had on your choices
5. No, please say why this message didn’t affect your choices
6. Did you find the informative message to be believable?
7. Yes
8. No
9. Why/ why not?

Additional questions to consider asking if the customer is engaged:

1. Why do you think you have not noticed the messages?
2. What do you focus on visually when you are at the cafeteria?
3. What are your priorities when making lunch choices at the cafeteria?
4. Have you observed your colleagues making more vegetarian choices?

**S2.** Mixed-effects regression model results for weekly total meal sales during the intervention period.

| **Variable** | **Model Estimate** | **Standard Error** | **95% CIs** | **t** | **p-value** |
| --- | --- | --- | --- | --- | --- |
|  | **Fixed Effects** | | | | |
| (Intercept) | 408.84 | 153.27 | [91.00, 726.68] | 2.67 | 0.014 |
| Trial Arm^[[1]](#footnote-1)^ (ref=Control) | -223.25 | 198.40 | [-634.72, 188.22] | -1.13 | 0.273 |
| Baseline total sales^[[2]](#footnote-2)^  (ref= Low) | 1227.85 | 232.10 | [746.51, 1709.18] | 5.29 | 0.000 |
| Length of Week^[[3]](#footnote-3)^  (ref= 5 Days) | -26.39 | 16.37 | [-58.73, 5.96] | -1.61 | 0.109 |
|  | **Random Effect** | | | | |
| (Intercept) | 238376.7 | 72047.24 | [131736.4, 430980.6] |  |  |

**S3.** Sensitivity analysis (conducted excluding two cafeterias with low intervention fidelity) mixed-effects regression model results for weekly percentage of meat-free meal sales during the intervention period.

| **Variable** | **Model Estimate** | **Standard Error** | **95% CIs** | **t** | **p-value** |
| --- | --- | --- | --- | --- | --- |
|  | **Fixed Effects** | | | | |
| (Intercept) | 22.60 | 1.72 | [19.00, 26.19] | 13.11 | 0.000 |
| Trial Arm (ref=Control) | -2.10 | 2.62 | [-7.56, 3.36] | -0.80 | 0.431 |
| Baseline meat-free sales  (ref= Low) | 10.76 | 2.66 | [5.21, 16.30] | 4.05 | 0.001 |
| Length of Week  (ref= 5 Days) | -1.19 | 1.18 | [-3.53, 1.15] | -1.01 | 0.316 |
|  | **Random Effect** | | | | |
| (Intercept) | 29.84 | 10.55 | [14.92, 59.65] |  |  |

**S4.** Sensitivity analysis (conducted excluding two cafeterias with low intervention fidelity) mixed-effects regression model results for weekly percentage of meat-free meal sales during the intervention period with interactions.

| **Variable** | **Model Estimate** | **Standard Error** | **95% CIs** | **t** | **p-value** |
| --- | --- | --- | --- | --- | --- |
|  | **Fixed Effects** | | | | |
| (Intercept) | 20.30 | 1.95 | [16.19, 24.41] | 10.39 | 0.000 |
| Trial Arm (ref=Control) | 2.66 | 4.22 | [-6.25, 11.58] | 0.63 | 0.537 |
| Length of Week  (ref= 5 Days) | -1.05 | 1.19 | [-3.39, 1.30] | -0.88 | 0.380 |
| Baseline meat-free sales  (ref= Low) | 15.51 | 3.69 | [7.73, 23.29] | 4.21 | 0.001 |
| **Intervention x High Baseline^[[4]](#footnote-4)^** | -10.11 | 5.11 | [-20.90, .68] | -1.98 | 0.065 |
| Cafeteria Worksite Type^[[5]](#footnote-5)^  (ref= Manufacturing) | 3.06 | 3.20 | [-3.69, 9.81] | 0.96 | 0.353 |
| **Intervention x Office^[[6]](#footnote-6)^** | -1.85 | 4.77 | [-11.91, 8.21] | -0.39 | 0.702 |
|  | **Random Effect** | | | | |
| (Intercept) | 24.81 | 9.70 | [11.53, 53.40] |  |  |

**S5.** Sensitivity analysis (conducted excluding two cafeterias with low intervention fidelity) mixed-effects regression model results for weekly total meal sales during the intervention period.

| **Variable** | **Model Estimate** | **Standard Error** | **95% CIs** | **t** | **p-value** |
| --- | --- | --- | --- | --- | --- |
|  | **Fixed Effects** | | | | |
| (Intercept) | 361.88 | 157.11 | [34.18, 689.58] | 2.30 | 0.032 |
| Trial Arm (ref=Control) | -162.20 | 212.87 | [-606.24, 281.84] | -0.76 | 0.455 |
| Baseline total sales  (ref= Low) | 1373.19 | 255.84 | [839.51, 1906.87] | 5.37 | 0.000 |
| Length of Week  (ref= 5 Days) | -13.84 | 17.34 | [-48.17, 20.48] | -0.80 | 0.426 |
|  | **Random Effect** | | | | |
| (Intercept) | 238376.7 | 72047.24 | [131736.4, 430980.6] |  |  |

1. Trial arm refers to whether a cafeteria was randomized to the control or intervention condition during the RCT. [↑](#footnote-ref-1)
2. Baseline meat free meal sales refer to whether a cafeteria had low (i.e. lower than the mean percentage of meat-free meal sales of all cafeterias) or high (i.e. higher than the mean percentage of meat-free meal sales of all cafeterias) percentage of meat-free meal sales during the eight-week period prior to the trial. [↑](#footnote-ref-2)
3. Length of week refers to the number of days the cafeteria was open and operational. Some cafeterias experienced closures due to strikes and till malfunctions which led them to be open four days of the week instead of five, and this was entered to the model as a dummy variable. [↑](#footnote-ref-3)
4. This interaction was entered into the model to predict the effect of a cafeteria that had a high baseline of meat-free meal sales being randomized into the intervention arm of the trial on its percentage of meat-free meal sales during the trial period. [↑](#footnote-ref-4)
5. Cafeteria worksite type refers to the nature of work being performed in the specific worksite that each cafeteria service. Worksites are either categorized as manufacturing (e.g. production and processing of various goods, distribution and storage facilities) or office (e.g. customer service call centers, financial and accounting services) sites. [↑](#footnote-ref-5)
6. This interaction was entered into the model to predict the effect of a cafeteria that was serving a worksite that was categorized as an office site being randomized into the intervention arm of the trial on its percentage of meat-free meal sales during the trial period. [↑](#footnote-ref-6)
